# Retention of strength and ion release of some restorative materials

**DOI:** 10.1007/s10266-024-01010-3

**Published:** 2024-09-26

**Authors:** Sufyan Garoushi, Timo Peltola, Minna Siekkinen, Leena Hupa, Pekka K. Vallittu, Lippo Lassila, Eija Säilynoja

**Affiliations:** 1 Department of Biomaterials Science and Turku Clinical Biomaterial Center-TCBC, Institute of Dentistry, University of Turku, Turku, Finland; 2 Research Development and Production Department, Stick Tech Ltd-Member of GC Group, Turku, Finland; 3 City of Turku Welfare Division, Oral Health Care, Turku, Finland; 4 Johan Gadolin Process Chemistry Centre, Åbo Akademi University, Turku, Finland

**Keywords:** Fiber-reinforced flowable composite, Flexural strength, Stability, Mineralization, Ion release

## Abstract

This study aimed to investigate the retention of strength in accelerated aging condition and ion release from an experimental fiber-reinforced bioactive flowable composite resin (Bio-SFRC), comparing it with various commercially available ion-releasing materials. The flexural strength of Bio-SFRC and other materials (Biodentine, TheraCal LC, Fuji II LC and Surefil one) was evaluated (*n* = 8) before and after hydrothermal accelerated aging. Ion concentrations of silica and phosphorus were measured after 1, 2, 3, 4, 7, 10, 14, and 21 days of specimen immersion in simulated body fluids (SBF) using UV–Vis spectrometry. In addition, ion release and pH change were studied in a continuous dynamic system in SBF over a period of 72 h. SEM and EDS were used to evaluate the microstructure on the top surface of the materials after SBF immersion. Data were statistically analyzed using variance ANOVA analysis (*p* = 0.05). Bio-SFRC showed higher flexural strength before (134.9 MPa) and after (63.1 MPa) hydrothermal aging compared to other tested materials (*p* < 0.05). Flexural strength significantly decreased after aging (*p* < 0.05) except for Fuji II LC which showed no significant differences. Ion release data showed that experimental Bio-SFRC slowly released phosphate ions. Biodentine and TheraCal LC had the strongest ability to form calcium phosphate precipitation on the material surface. Phosphate ion release cannot be detected clearly from these materials. Surefil one and Fuji II LC were more stable materials without any observable ion release. The advantages of fiber containing structure and slow release of ions suggest that experimental Bio-SFRC is a promising bioactive material to provide ions for mineralization of surrounding tissues, and keeping the durability of the materials at higher level than that of other tested materials.

## Introduction

In the context of the biomimetic concept, contemporary dentistry seeks inspiration from nature by examining the biology, structure, and function of the tooth as a blueprint for developing novel or enhanced materials and methods. The goal is to replace or restore teeth in a biomechanically appropriate manner through design and engineering [1].

From a biomechanical perspective, the endeavor is to restore lost tooth structure using biomaterials that mirror comparable mechanical and physical characteristics, with a focus on flexural strength and fracture toughness [1, 2]. There are several options to replace the lost dentine when constructing direct restorations in deep cavities. These consist of calcium silicate-based materials, glass ionomer cements (GICs), resin-modified GICs (RMGICs), conventional composite resins, and short fiber-reinforced composite resins [3, 4].

Compared to dentine, the fracture toughness of conventional particulate-reinforced composite resin, calcium silicate-based material, or GIC is still much lower, and their microstructure is not similar to that of dentine [5–8]. These materials comprise particles of filler packed within a polymeric matrix, whereas dentine is composed of collagen fibers enclosed in a hydroxyapatite matrix. Hence, it is more accurate to consider dentine as a natural fiber-reinforced composite [1]. Dentine is given unique properties by collagen fibers, which act as crack stoppers and simultaneously make the material durable, flexible, and tough [9]. Therefore, significant enhancements could be achieved by utilizing a composite resin with high toughness that closely mimics the properties of dentine as a substitute.

Strengthening the composite resin and GIC by incorporating short glass fibers is shown to be a highly effective strategy among the recently investigated methods [10–13]. Incorporated short fibers improved the material’s resistance to crack propagation and concurrently decreased the stress intensity at the crack tip, preventing the unstable spread of cracks. Thus, the fibers increased the material’s toughness and the fracture resistance [2].

On the other hand, the focus of restorative dentistry has steadily switched from emphasizing biocompatibility to prioritizing bioactivity. The scientific and clinical focus on bioactive restorative materials is expanding in line with the development of minimally invasive and biologically oriented dental treatment approaches. Nevertheless, the term “bioactive materials” or “bioactivity” remains a topic of debate [14]. The definition of bioactivity, according to the critical evaluation by Mocquot et al., relies on the clinical application, ranging from the ability to produce apatite reprecipitation on the surface of enamel and dentine to cellular effects carried on by the release of bioactive molecules and ions [15]. This definition agrees with the statement of the FDI World Dental Federation Policy that outlines in 2022 which defines a bioactive dental restorative material as one that can function chemically, biologically, or by combining the two modes [16].

Yet, research indicates that the brittleness and low fracture toughness of contemporary bioactive or ion-releasing materials used as dentine substitutes, cavity liners, or bases still pose problems, especially when utilized in large restorations [4, 5]. The inclusion of such non-silanized bioactive particles lack reinforcement capabilities seen in silanized glass particles or fibers, their presence does not contribute to strengthening. Moreover, the dissolution of bioactive particles could potentially have adverse effects on the material’s long-term mechanical stability [17]. From clinical perspectives, a complete understanding of the use of these bioactive materials overhead their biomechanics limitations and elsewhere their specific indications would result in restoration failure and the loss of natural dental tissue [18].

Designing restorative materials with integrate specific bioactive properties to fulfill the demands of the biomechanics and bioactivity concept represents a challenging endeavor. As far as our knowledge extends, dentistry currently lacks a material that can release ions while maintaining mechanical stability. Hence, our research aimed to characterize the mechanical stability and ion release of an experimental fiber-reinforced ion-releasing flowable composite resin (Bio-SFRC), comparing it with various commercially available ion-releasing materials.

The suggested null hypotheses were: (1) there is no difference between tested materials in mechanical properties before and after aging, (2) there is no difference between tested materials in ion release profile at different time points.

## Materials and methods

Table 1 provides comprehensive details of all materials employed in this investigation.

**Table 1:** Materials used in the study

Brand (code)	Manufacturer	Type	Composition
Biodentine	Septodont, France	Self-cured tricalcium silicate material	Powder: tricalcium silicate, dicalcium, silicate, calcium carbonate, oxide fillers (79 wt%) Liquid: water, calcium chloride, and hydro-soluble polymer
TheraCal LC	Bisco Inc, USA	Light-cured tricalcium silicate material	Polyethylene glycol dimethacrylate, Portland cement type III, fumed silica, barium zirconate (55 wt%)
Fuji II LC	GC Corp, Japan	Light-cured resin-modified glass ionomer	Powder: fluoro-aluminum silicate glass (76 wt%) Liquid: HEMA, Polyacrylic acid and water
Surefil one	Dentsply Sirona, USA	Self-cured composite hybrid	Powder: silanated aluminum phosphor–strontium–fluoro-silicate glass, silicon dioxide, ytterbium fluoride (77 wt%) Liquid: acrylic acid, polycarboxylic acid, bifunctional acrylate, water
Bio-SFRC	Experimental	Light-cured fiber-reinforced flowable composite	UDMA, dimethacrylate monomers Borosilicate glasses containing TiO2 and ZnO. Carbonated apatite and calcium carbonate particles (62 wt%)

UDMA, urethane dimethacrylate; HEMA, 2-hydroxyethyl methacrylate; wt%, weight percentage of inorganic fillers

### Mechanical test

Specimens for the three-point bending test (2 × 2 × 25 mm^3^) were fabricated from each material. Bar-shaped specimens were fabricated using half-split stainless steel molds and transparent Mylar sheets. Materials were manipulated according to manufacturer instructions. The light-cured materials were photoactivated using a handheld light-curing device (Elipar LED, TM S10, 3 M ESPE, Germany) for 20 s in five overlapping sections on both sides of the metal mold. The light had a wavelength ranging from 430 to 480 nm, and the average light intensity was 1600 mW/cm^2^ (MARC^®^ Resin, BlueLight analytics Inc., Halifax, Canada). The materials were left in their molds for 30 min before being carefully removed. The specimens from each material (n = 8) were subjected to one of two conditions: either stored dry for one day at 37 °C or boiled in deionized distilled water for 16 h prior to testing. The 3-point bending test was conducted in accordance with the ISO 4049 standard, with a test span of 20 mm, a cross-head speed of 1 mm/min, and an indenter diameter of 2 mm. All specimens were loaded into a material testing machine (model LRX, Lloyd Instruments Ltd., Fareham, England), and load–deflection curves were recorded using PC-computer software (Nexygen 4.0, Lloyd Instruments Ltd., Fareham, England). Flexural strength (FS) was calculated using the following formula:

$${\text{FS}} = {\text{ 3F}}_{\text{m}} {\text{I}}/\left( {{\text{2bh}}^{2} } \right)$$

where *F*_*m*_ represents the applied load (*N*) at the highest point of the load–deflection curve, *I* denotes the span length (20 mm), and *b* and *h* represent the width and thickness of the test specimens, respectively.

### Ion release and pH measurements

A simulated bodily fluid (SBF) was used for the in vitro ion release experiments [19, 20]. The measurement of ion release was conducted utilizing two separate approaches, namely static and dynamic. For the static approach, three disk-shaped specimens (5 mm in diameter and 2 mm in thickness) were prepared from each studied material using a metal mold. The material was inserted into the mold and then covered on both sides with Mylar strips and microscopic glass slides to remove any extra material. The manipulation of materials was carried out as previously stated. Subsequently, these specimens were immediately utilized to evaluate the materials’ reactions in SBF.

In a closed polyethylene container, each disk was submerged in 40 ml of SBF. As controls, three SBF samples that were kept in empty containers were used to assess the stability of the solution. Ion concentrations of silica (*n* = 9/material) and phosphorus (*n* = 9/material) were assessed after 1, 2, 3, 4, 7, 10, 14, and 21 days of specimen immersion in SBF. The molybdenum blue method was used to measure the amounts of phosphorus using a UV–Vis spectrophotometer (Specord 200 Plus, Analytik Jena, Germany). The phosphorus analysis was conducted using the Lowry–Lopez method [21]. The amount of silicon released into SBF was used to calculate the differences in solubility of several materials. The concentration of silicon in the sample solutions was tracked as a function of immersion time. The UV–Vis spectrophotometer was also used to examine the silicon concentrations. Based on reduction with 1-amino-2-naphthol-4-sulfonic acid, the silicon analysis was conducted [22].

**Fig. 1:**
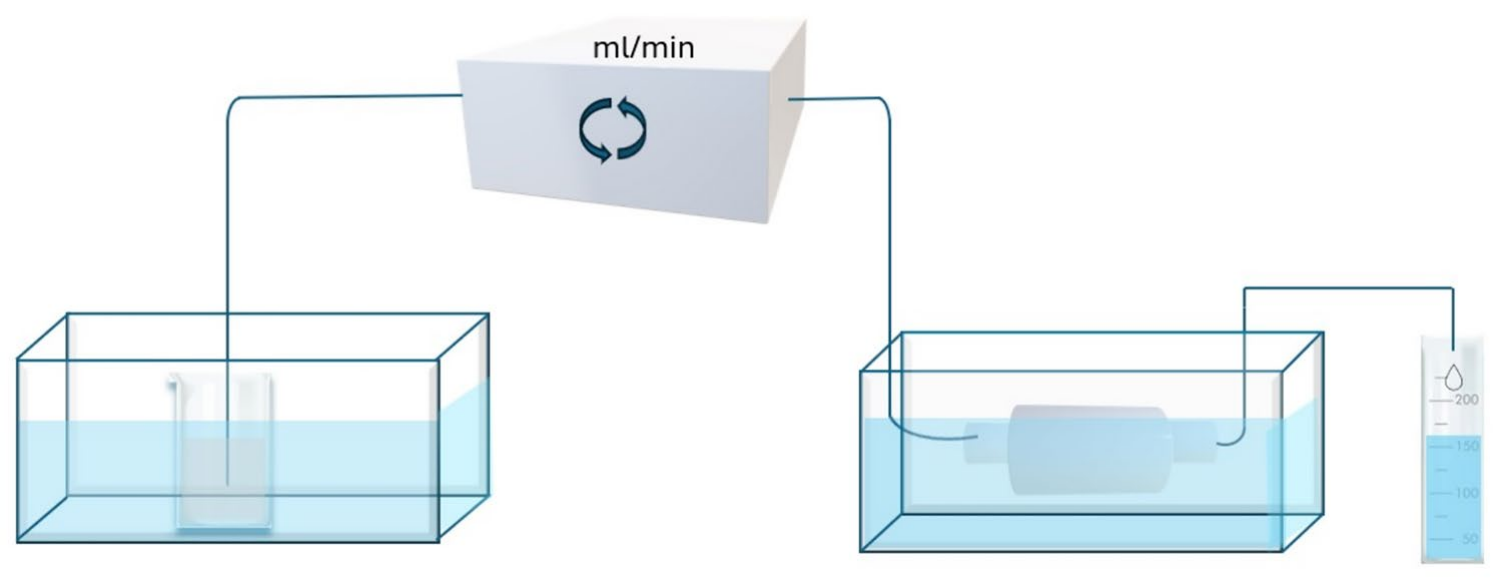
Test setup for dynamic continuous flow approach

For the dynamic continuous flow approach, cylinder-shaped (3 mm in diameter and 8 mm in length) specimens were made (*n* = 3/material) and then immediately placed in a reactor (Fig. 1). SBF was fed through the reactor for 72 h with a peristaltic pump (0.04 ml/min). Ion concentrations in the outflow were measured at different time points (0, 2, 5, 10, 24, and 72 h) with inductively coupled plasma optical emission spectroscopy (ICP-OES, PerkinElmer Optima 5300 DV, Waltham, MA, USA). The pH was measured for all the collected outflow solutions (2.4 ml) collected at each time point (0, 1, 2, 5, 10, 24, 48, 72 h). The pH was measured at room temperature using two different pH meters (Mettler Toledo SevenEasy or VWR pHenomenal pH 1100 L). Then, using the following equation, the pH values recorded at room temperature were converted to pH at 37 °C [23].

### Microscopic analysis

Scanning electron microscopy (SEM) and energy-dispersive spectroscopy (EDS) (TM4000Plus, Hitachi, Japan) were used to characterize the microstructure and perform chemical analysis of the examined specimen surfaces after immersion in SBF. In addition, EDS analysis was also performed before SBF immersion.

### Statistical analysis

To determine the differences between the groups, the data were statistically analyzed using SPSS version 23 (SPSS, IBM Corp.) and analysis of variance (ANOVA) at the *p* < 0.05 significant level. A Tukey HSD post hoc test was then performed.

## Results

**Fig. 2:**
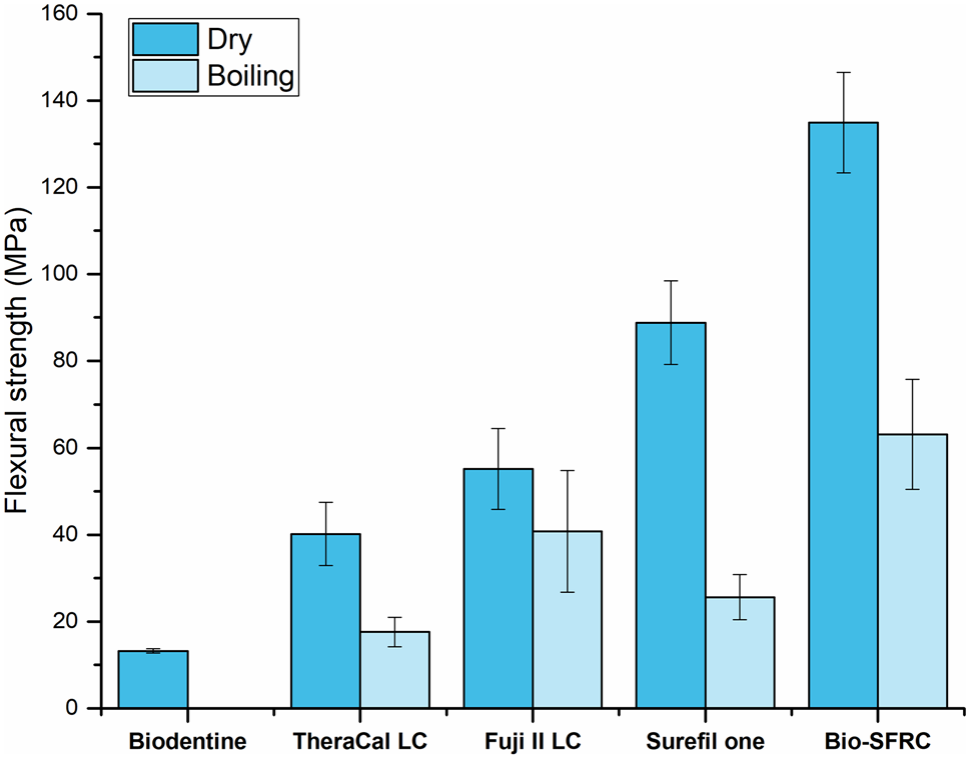
Mean flexural strength (MPa) and standard deviations (SD) of tested materials before and after hydrothermal aging (boiling in water for 16 h). Biodentine specimens could not be measured after aging as they had already fractured

As depicted in Fig. 2, Bio-SFRC outperformed the commercially evaluated materials in terms of flexural strength values both before (134.9 MPa) and after (63.1 MPa) hydrothermal aging (*p* < 0.05). Biodentine demonstrated the lowest flexural strength values (13.2 MPa), and specimens could not be tested after hydrothermal aging as they had already fractured. Except for Fuji II LC, which showed no significant differences, all materials (50–70%) had a significant decrease in flexural strength following aging (*p* < 0.05).

**Fig. 3:**
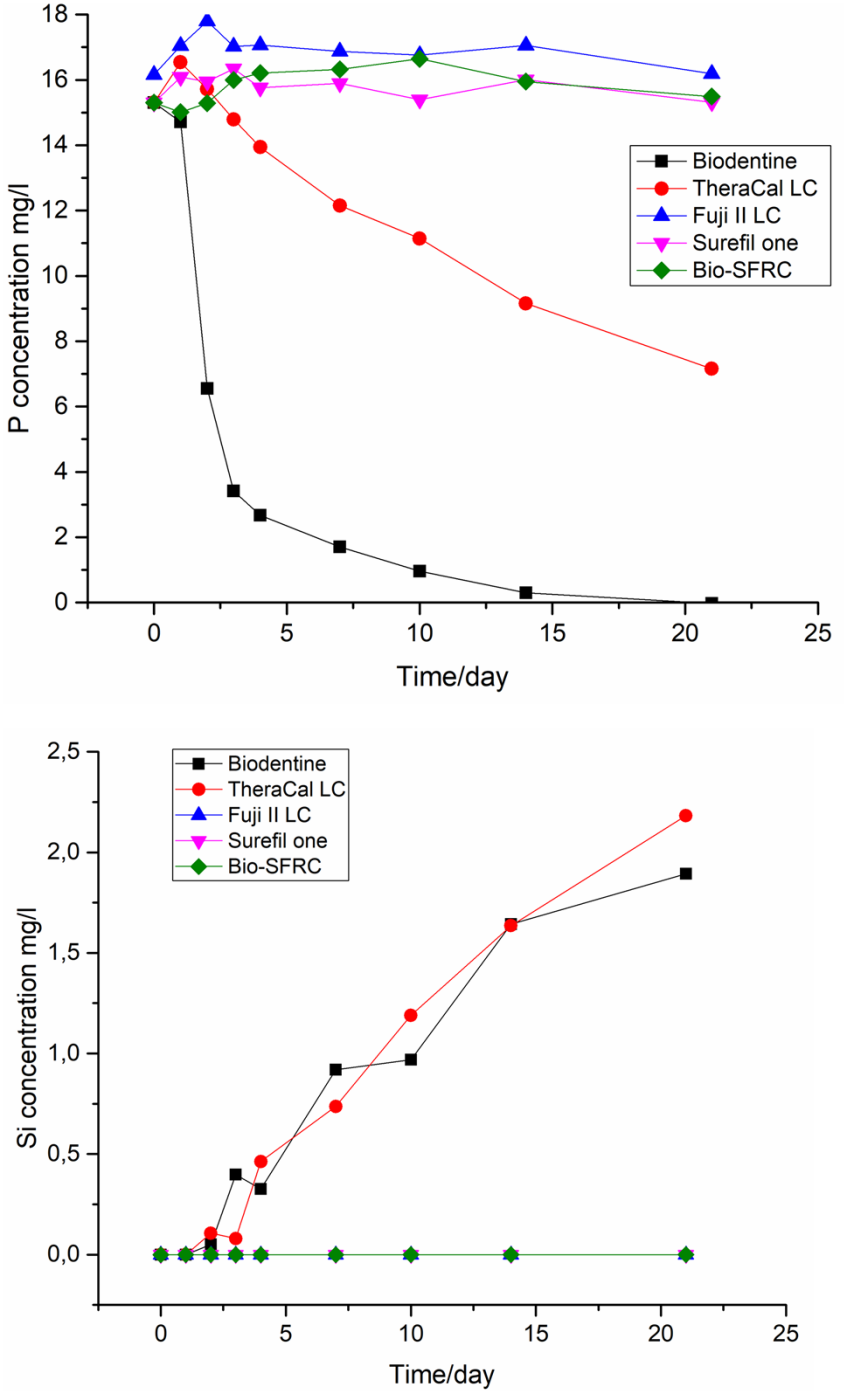
Cumulative concentrations of ions dissolved from the materials in static SBF for 3 weeks

Figure 3 presents the cumulative phosphorus and silica release (static approach) for the tested materials during a 3 weeks storage period in SBF. Ion analysis showed clear differences between the materials. The highest silica releases were measured for Biodentine and TheraCal LC. No significant difference was found between the other materials tested. Interestingly, Biodentine and TheraCal LC, i.e., the two samples that showed a minor Si release, also suggested P precipitation compared to around 16 mg/l measured for the control samples).

**Fig. 4:**
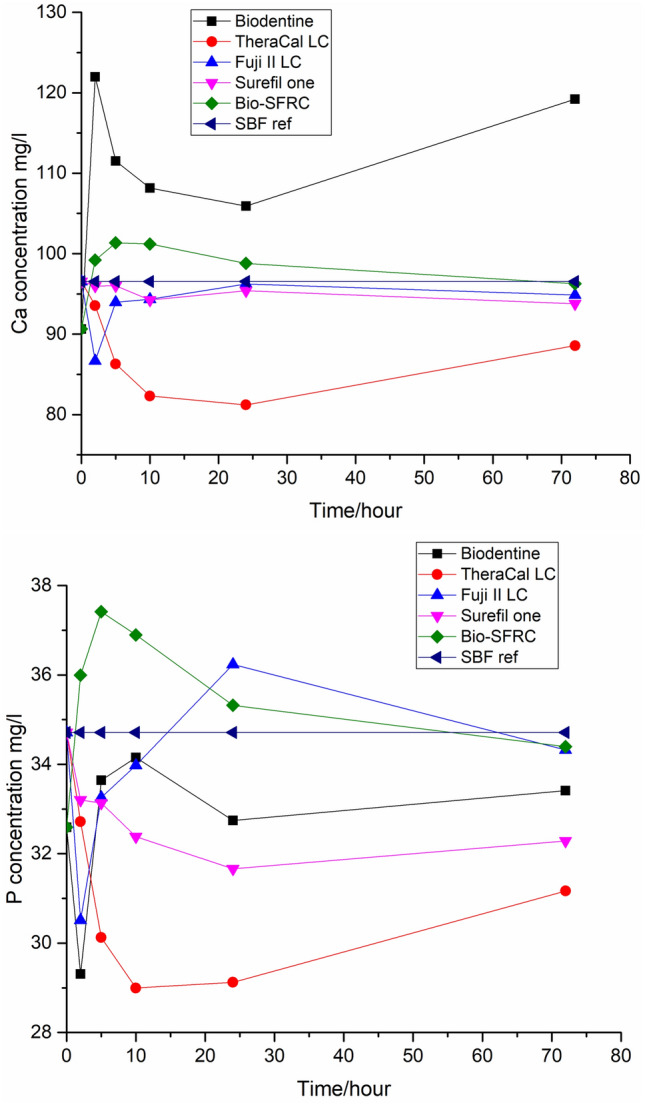
Concentrations of Ca and P ions (ICP-OES) released into continuous flow of SBF

**Fig. 5:**
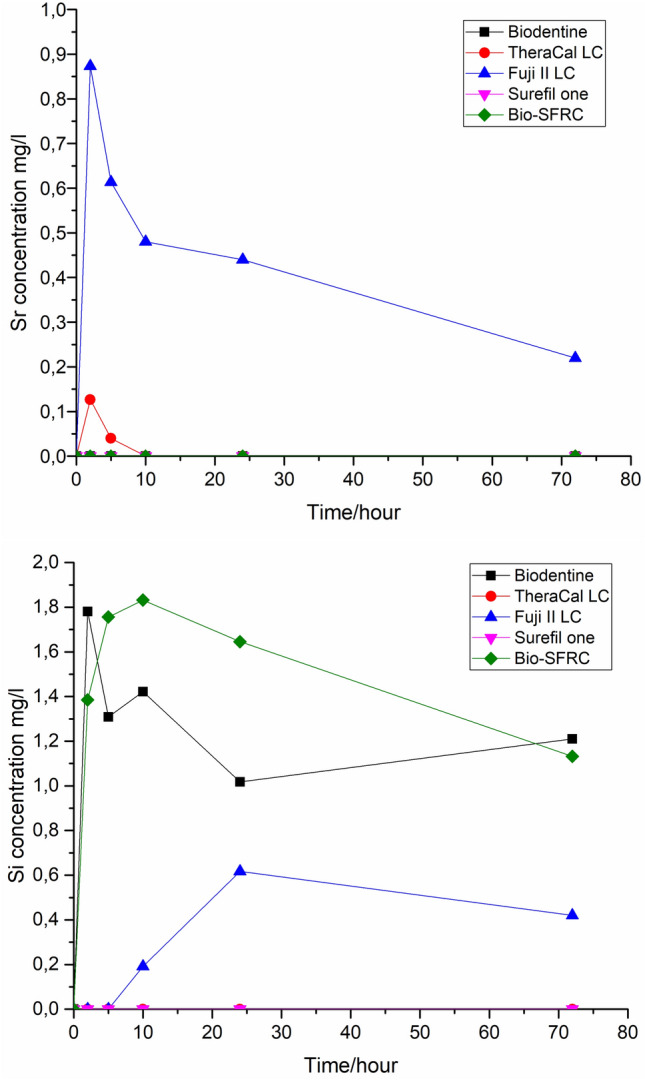
Concentrations of Sr and Si ions (ICP-OES) released into continuous flow of SBF

Figures 4 and 5 show concentrations of ions (calcium, phosphorus, silica and strontium) released from the samples into the continuous dynamic flow of SBF. Biodentine demonstrated the most prominent release of calcium ions. There is no significant difference observed among the other materials. However, both Ca and P concentrations are lowest for TheraCal LC. Some Si was released into the continuous flow of fresh SBF, i.e., a solution containing no Si, from Biodentine, Bio-SFRC and Fuji II LC (Fig. 5). Fuji II LC showed a minor release of Sr, while a very minor Sr release was measured for TheraCal LC.

**Fig. 6:**
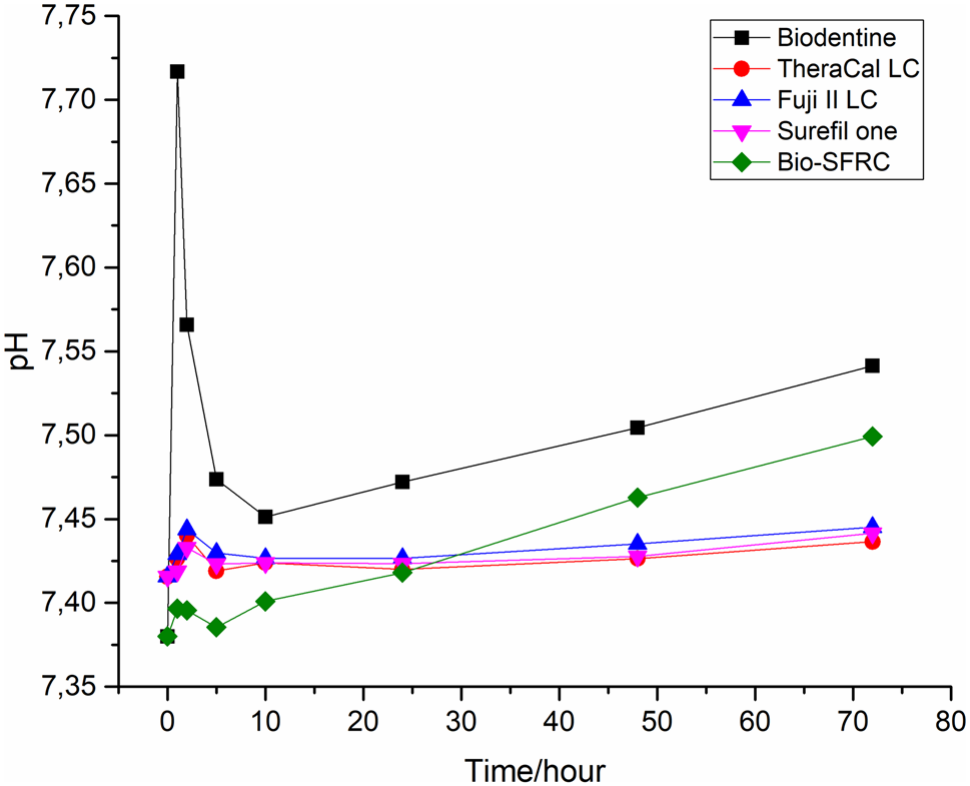
pH changes caused by ion release during continuous flow of SBF

In the dynamic continuous flow, the pH of SBF stayed around the initial value of 7.4 for the dissolution of Fuji II LC, TheraCal LC and Surefil one (Fig. 6). In contrast, the pH for the fresh SBF fed through Biodentine and Bio-SFRF steadily increased, suggesting that these materials gradually dissolved.

The chemical compositions per element (weight percentage) of each investigated material (before SBF immersion), as determined by EDS, are presented in Table 2. The main elements C, O, and Si were detected in all materials. A substantial amount of Ca was detected in Biodentine (40%), whereas a small amount (0.4%) was detected in Surefil one and Fuji II LC. The latter has the highest amount of F among the investigated materials. P was detected only in Bio-SFRC (5.5%) and Surefil one (2.3%). In addition, the heavy element Zr was identified in Biodentine and TheraCal LC, while Sr was identified in Fuji II LC and Surefil one (Table 2).

**Table 2:** Chemical constituents of investigated materials determined with EDS analysis before SBF immersion

Material	Weight %
Biodentine	C 13.1, O 32.1, Ca 40.8, F 0.3, Al 0.4, Si 9.2, Zr 4.1
TheraCal LC	C 20.5, O 43.4, Ca 11.5, Al 2.5, Si 13.5, N 4.5, Zr 1.3, Ba 2.8
Fuji II LC	C 14.0, O 32,0, Na 1.0, F 11.0, Al 12.4, Si 11.2, Ca 0.4, Sr 17.7, Ba 0.3
Surefil one	C 29.7, O 27.1, Na 2.7, F 5.8, Al 8.9, Si 9.6, Cl 5.4, K 0.5, Ca 0.4, Yb 3.3, P 2.3, Sr 4.3
Bio-SFRC	C 23.8, O 38.5, Na 1.8, F 2.2, Mg 0.1, Al 2.6, Si 9.0, P 5.5, K 0.6, Ca 14.7, Ti 0.5, Zn 0.7

**Fig. 7:**
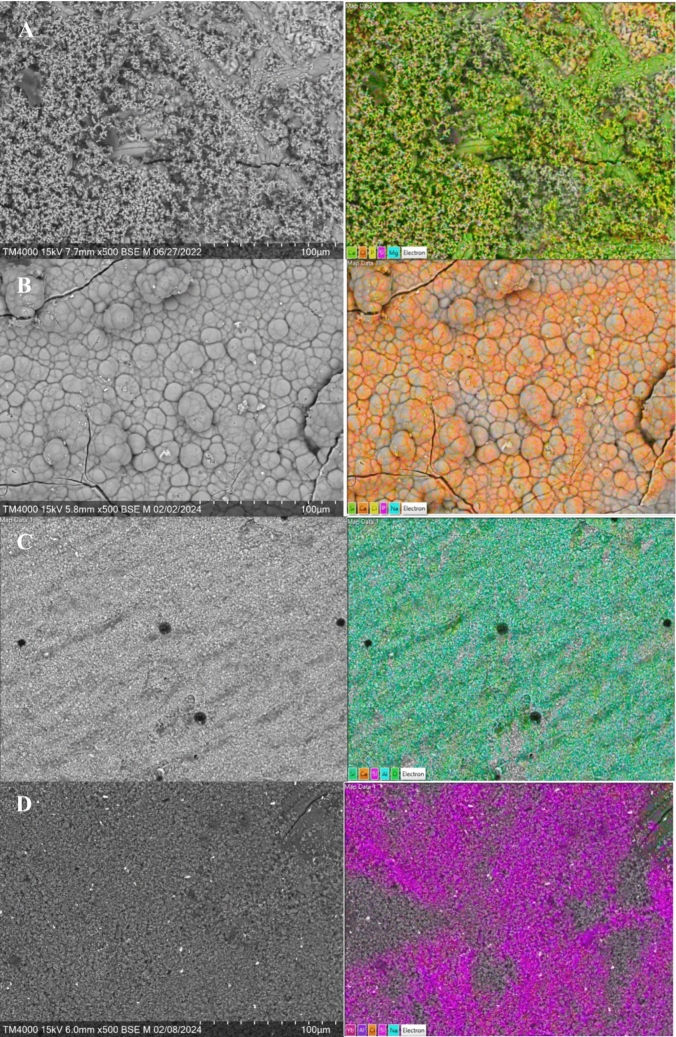

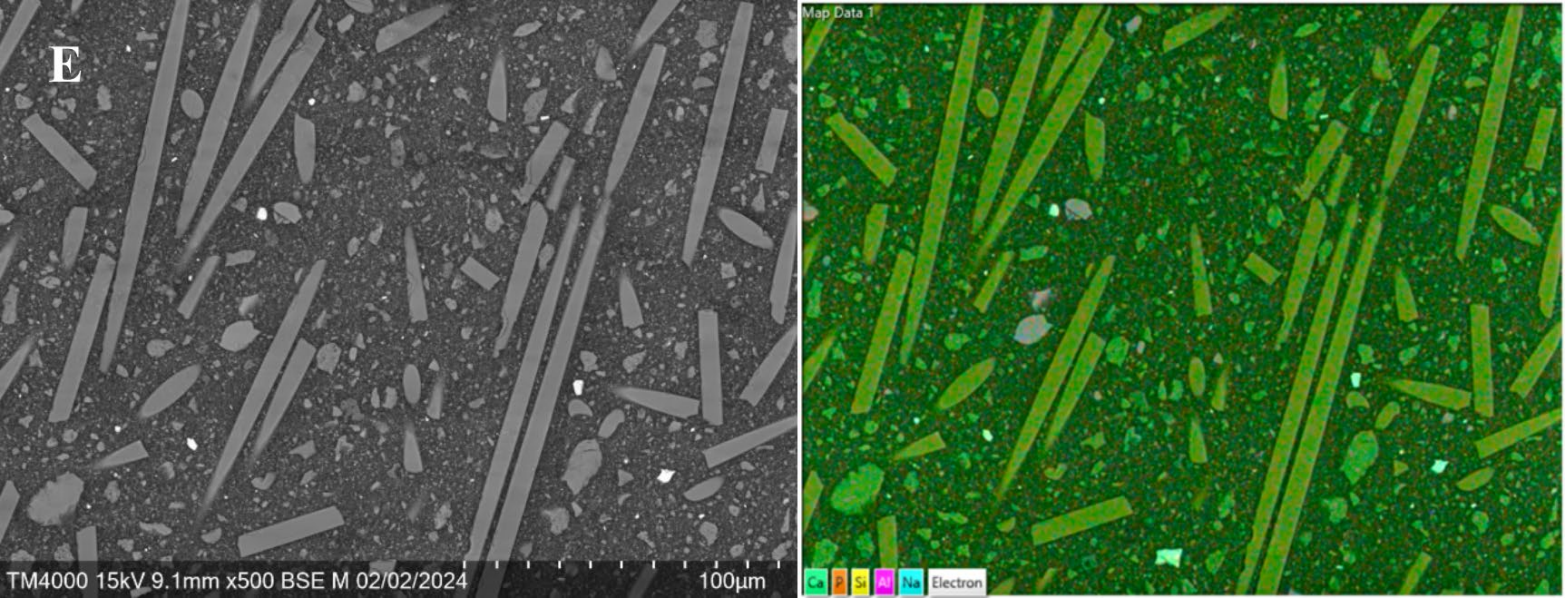
SEM images (left) with an elemental mapping analysis (right) of the material surfaces after 3 weeks in SBF. **A** Biodentine; **B** TheraCal LC; **C** Fuji II LC; **D** Surefil one; **E** Bio-SFRC

SEM micrographs and EDS analyses of the material surfaces after 21 days in static SBF are shown in Fig. 7. TheraCal LC shows a distinct calcium phosphate (CaP) layer and enrichment of Ca (Fig. 7B). The surface layer structure and Ca and P analyses also suggest some CaP on Biodentine (Fig. 7A). Although typical CaP structure cannot be observed on Bio-SFRC, calcium and phosphate were enriched on the surfaces (Fig. 7E). No distinct surface layer or enrichment of calcium and phosphate were detected on Surefil one (Fig. 7D) and Fuji II LC (Fig. 7C).

## Discussion

Minimally invasive restorative dentistry requires the development of new bioactive materials that can withstand the forces of mastication without fracturing, endure the effects of hydrothermal aging in the mouth, are mechanically stable and release ions support biomineralization. This work evaluated/compared the mechanical stability and ion release profile of fiber-reinforced bioactive flowable composite resin (Bio-SFRC) with commercially available ion-releasing materials. The null hypotheses proposed in this investigation were rejected as significant differences were seen in the retention of strength and ion release profile between the materials investigated.

The flexural strength values obtained were comparable to those reported in the literature [7, 8, 24, 25]. The results confirm/suggest that microfibers, i.e., fibers with a length scale of 200–300 μm and a diameter of 6 μm found in Bio-SFRC, can significantly affect/increase the flexural strength and mechanical stability of a high-flowable ion-releasing material while maintaining its user-friendly features. The microfiber length in the flowable Bio-SFRC was less than the reported critical fiber length [8]. The aspect ratio exceeded 30, providing enhanced reinforcement for the material and successfully absorbing stress from the matrix. According to the literature, the fracture toughness of Bio-SFRC is 1.6 MPa m^1/2^, whereas for Surefil one and Fuji II LC, it ranges between 0.3 and 0.8 MPa m^1/2^ [7, 8, 26].

This study also examined the impact of hydrothermal accelerated aging, specifically boiling, on the flexural strength of the materials. Although laboratory hydrothermal aging may not precisely correspond to the clinical condition, it offers insight into the long-term stability of materials, as suggested by previous research [27, 28]. The data confirmed the prior finding that boiling dental composite in water can rapidly allow water to enter the composite structure, resulting in the softening of the composite matrix [28]. In addition, it has been suggested that the infiltrated water plays a role in the breakdown of non-silanized bioactive particles by hydrolytic degradation. This leads to the hydrolysis of the interface between the particles and the matrix, causing an increase in the softness of the matrix [17]. Therefore, the decrease in the flexural strength of the tested materials following hydrothermal aging were attributed to the above-mentioned processes. Previous investigations have indicated that the strength of glass ionomer materials does not deteriorate when exposed to water, regardless of the presence or absence of resin reinforcement [29]. The increased maturation of the cement matrix over time may provide a potential explanation for the observed performance of Fuji II LC following hydrothermal aging, as illustrated in Fig. 2.

Biodentine is based on tricalcium silicate and undergoes a hydration process. It tends to absorb water that can cause swelling and degradation of the cement structure, weakening of its mechanical properties [30]. Hence, the reduction in strength is also probably due to some hydrolysis of the material components. After hydrothermal aging, all Biodentine specimens had pre-test failure, probably due to swelling, as the process of boiling can rapidly allow water to enter the cement structure. As stated by Bachoo et al., the crystallization process of Biodentine persists for a duration of up to 2 weeks [31]. It reaches a stage of bulk set during this period, accompanied by enhancements in its mechanical properties. This observation could explain the pre-test failure patterns observed following hydrothermal aging.

The ion dissolution analyses revealed distinct differences between the materials. In continuous SBF flow, some calcium and phosphate ions were dissolved from Bio-SFRC, as shown by the slightly higher concentrations than in the inflow during the 72 h test (Fig. 5). Most likely, the presence of functional bioactive fillers, specifically calcium carbonate and carbonated apatite (Table 1), which make up more than 20% of the content of Bio-SFRC, contributed to the releases [32, 33]. Moreover, the release of silicon ions suggests that the composite components slowly dissolve. The polymer matrix in Bio-SFRC gradually absorbs water, facilitating ion diffusion, thus preventing their entrapment inside the material [8]. Furthermore, a recent investigation indicated that exposed short fibers may contribute positively to ion leaching from the cross-linked composite structure [34]. Despite the ion release from Bio-SFRC, no distinct calcium phosphate (CaP) layer was analyzed on the material after 3 weeks in SBF (Fig. 7). It was assumed that the ion release from the material and the sample surface area to SBF volume ratio did not support the formation of a thick mineralized layer on cross-linked composite surfaces as proven in previous research [8, 35].

The rapid decrease of calcium ion concentration and increase of silicon ion concentration from Biodentine during the static immersion in SBF (Fig. 3) align with the thick CaP layer seen on the sample surface after 21 days (Fig. 7). Calcium and phosphate ion concentration patterns in the continuous SBF flow also support a high bioactivity (Fig. 4). Calcium was continuously released, as shown by the concentration values higher than in the SBF inflow at all time points. Simultaneously, the concentration of phosphorus was less than that in the inflow, thus suggesting a steady consumption by CaP precipitation. These results are consistent with several investigations in the literature, which consistently report that Biodentine has a higher calcium release rate and quicker apatite formation than other materials used for pulp-capping [36, 37]. In addition, the other calcium silicate-based material, TheraCal LC formed CaP precipitate on the material surface, as evidenced by the notable decrease in the phosphorus concentration in static SBF dissolution (Fig. 3) and SEM/EDS analysis of the surface (Fig. 7). Also for TheraCal LC, the low phosphorus concentrations in the continuous SBF flow suggest that it has reacted with calcium to form CaP. In addition, the steady but minor increase of silicon in the static dissolution from TheraCal LC and Biodentine suggests that these materials degraded slowly (Fig. 3).

The measured steady increase of the pH of SBF upon the continuous dissolution of Biodentine and Bio-SFRC was assumed be due to calcium release. The gradually increasing solution pH upon feeding fresh SBF along the samples might correlate with some beneficial effects when using these materials in restorative dentistry, e.g., antimicrobial properties and promotion of pulp cell activity for repair processes [14, 15]. In addition, an alkaline pH environment can help balance off acidic conditions in the mouth, which is advantageous for protecting tooth structure and supporting remineralization [15].

Yao et al., compared the structure and chemical properties of Surefil one and Fuji II LC [38]. They reported substantial amounts of phosphate in Surefil one (16 wt%) but not in Fuji II LC. According to the ion release data in the present study, both Surefil one and Fuji II LC were stable in SBF, due to minor ion release measured. In accordance, no distinct CaP layer was identified on any of them (Fig. 7). Our findings align with several laboratory investigations that have failed to demonstrate the mineralizing capacity of glass ionomer-like materials [8, 39, 40].

Fuji II LC showed a small burst of strontium ion release (Fig. 5). Fuji II LC is a resin-modified glass ionomer cement (GIC) containing aluminum, fluorine, and strontium as part of the GC’s classical GIC filler (Table 2). Strontium is known to provide antibacterial activity [41]. Fluorine release was not measured in this study but it has been already demonstrated through several studies [6, 7, 24]. Fuji II LC contains more fluorine than Surefil one [38].

Considering that fluid flow in vivo is minimal between restorative material and dentine, the static approach to measuring ion release is rational, simpler, and more straightforward to perform compared to the dynamic approach. However, the latter simulates more closely the dynamic conditions present in the oral cavity, including the continuous flow of saliva and other fluids.

Although the current investigation yielded useful information, it was limited by the absence of ion analysis for elements proved to have clinical benefits such as fluorine and zinc. Furthermore, a more comprehensive assessment of the cytocompatibility and antibacterial effect of these materials is required.

## Conclusion

Bio-SFRC, an experimental light-cured fiber-reinforced flowable composite, had higher flexural strength values before and after hydrothermal aging compared to several commercial ion-releasing materials. The advantages of its fiber containing structure and slow release of ions suggest that Bio-SFRC is a promising bioactive material to provide ions for mineralization of surrounding tissues, and keeping the durability of the materials at higher level than that of other tested materials.

## Data Availability

The data presented in this study are available on reasonable request from the corresponding author.
